# Prediction of Drug–Target Interactions From Multi-Molecular Network Based on Deep Walk Embedding Model

**DOI:** 10.3389/fbioe.2020.00338

**Published:** 2020-06-03

**Authors:** Zhan-Heng Chen, Zhu-Hong You, Zhen-Hao Guo, Hai-Cheng Yi, Gong-Xu Luo, Yan-Bin Wang

**Affiliations:** ^1^The Xinjiang Technical Institute of Physics and Chemistry, Chinese Academy of Sciences, Urumqi, China; ^2^University of Chinese Academy of Sciences, Beijing, China; ^3^School of Cyber Science and Technology, Zhejiang University, Hangzhou, China

**Keywords:** drug–target interactions, molecular association network, attribute feature, behavior feature, random forest

## Abstract

Predicting drug–target interactions (DTIs) is crucial in innovative drug discovery, drug repositioning and other fields. However, there are many shortcomings for predicting DTIs using traditional biological experimental methods, such as the high-cost, time-consumption, low efficiency, and so on, which make these methods difficult to widely apply. As a supplement, the *in silico* method can provide helpful information for predictions of DTIs in a timely manner. In this work, a deep walk embedding method is developed for predicting DTIs from a multi-molecular network. More specifically, a multi-molecular network, also called molecular associations network, is constructed by integrating the associations among drug, protein, disease, lncRNA, and miRNA. Then, each node can be represented as a behavior feature vector by using a deep walk embedding method. Finally, we compared behavior features with traditional attribute features on an integrated dataset by using various classifiers. The experimental results revealed that the behavior feature could be performed better on different classifiers, especially on the random forest classifier. It is also demonstrated that the use of behavior information is very helpful for addressing the problem of sequences containing both self-interacting and non-interacting pairs of proteins. This work is not only extremely suitable for predicting DTIs, but also provides a new perspective for the prediction of other biomolecules’ associations.

## Introduction

Prediction of drug–target interactions (DTIs) is one of the most important steps in the genomic drug discovery pipeline and drug repurposing (Knowles and Gromo, 2003; Yildirim et al., 2007), the purpose is to discover putative new drugs and new uses of existing drugs. To our knowledge, the effects of many useful protein targets on drugs are modulated by interacting with ligands, including enzymes, ion channels, G protein-coupled receptors and nuclear receptors (Yamanishi et al., 2010). The development of rapid sequencing technology and the implementation of the human genome project, which has produced massive amounts of biological data, has given birth to a new discipline—computational biology. Before this, many traditional biological experimental methods were used to discover the relationships between proteins. Such as Co-immunoprecipitation (CO-IP), Tandam affinity purification (TAP), Glutathione-S-transferase (GST) pull down, phage display technology, yeast two-hybrid, and so on. However, due to the limitation of flux, precision and cost, it is often difficult to realize large-scale DTIs using traditional biological experimental methods. Therefore, computer-assisted methods are increasingly used in DTI predictions, and provide an effective means for the discovery and screening of lead compounds.

Recently, several computational methods were developed and considered to discover the DTIs (Chen et al., 2015; Chan and You, 2016; Luo et al., 2017). Many researchers have made great efforts to develop useful algorithms to deal with various DTI-related prediction problems. The most commonly used algorithms are docking simulations, literature text mining, machine learning, and network information, among others. Luo et al. (2017) proposed a network integration method for DTI detection and computational drug repositioning from heterogeneous information. Wong et al. (2015) analyzed the docking modes of 20 drugs and 28 proteins, and determined that 13 drugs could target 11 proteins at the same time, and designed multi-target drug complexes to destroy the mechanism of action of various cancers. Heinemann et al. (2016) systematically analyzed publication patterns appearing along the drug discovery process of targeted cancer therapies in the literature, and provided a support tool for novel drug development. Mayr et al. (2018) obtained different types of molecular descriptors on a ChEMBL dataset, and made a wide range of comparison with several machine learning models for detecting DTIs. Lu et al. (2019), based on the assumption that similar drugs share similar patterns of relationships with target proteins, proposed a heterogeneous network embedding model to predict DTIs by integrating the drug–drug similarity network, target–target similarity network and known DTIs into a heterogeneous network, called HNEDTI. Zhang et al. (2019) introduced how to calculate similarities based on drug–drug similarity and target–target similarity, and summarized, analyzed, and compared different machine learning-base prediction models. Based on these methods, we proposed a multi-molecular network, also called molecular associations network (MAN; Guo et al., 2019) to detect the interactions between drug candidates and related target proteins.

In the MAN, we not only used DTI data, but also added other biomolecules’ interactions information in the network. The main idea of this work comes from computational systems biology (Kitano, 2002; Materi and Wishart, 2007), network biology (Barabasi and Oltvai, 2004; Emmert-Streib and Glazko, 2011; Cahan et al., 2014), and network representation learning (Yang et al., 2015; Zhang et al., 2018). Computational systems biology aims to reveal new biological characteristics from a systematic perspective and use interdisciplinary tools to integrate and analyze large amounts of complex heterogeneous data from various experiments. It plays a key role in many complex processes occurring in biological systems. Subsequently, as more and more large and diverse data were collected at multiple levels of the system biology, Barabasi and Oltvai (2004) proposed network biology to understand the cell’s functional organization. Network biology refers to studying the biosystem network using mathematical methods and graph theory, and the network topology model. The studies have shown that cellular networks obey the general rules of network science, and it is helpful for understanding the interactions between molecules inside a living cell. Afterward, inspired by deep learning and word embedding technology in natural language processing (NLP), vector representation of nodes in automatic learning networks has become a research hotspot (Goldberg and Levy, 2014; Pennington et al., 2014; Peters et al., 2018; Devlin et al., 2018; Yang et al., 2019). This work has been gradually applied to the field of bioinformatics.

To summarize, Guo et al. (2019) for the first time proposed a MAN by integrating the associations among miRNA, lncRNA, protein, drug, and disease, where any kind of potential associations can be predicted. In this paper, we constructed a biomolecular relationship network, which contains nine kinds of associations with five types of molecules. All the molecules in the MAN were treated as nodes and all the relationships were regarded as edges. The associations between a node and other nodes in the complex network were called the behavior of the node. This work introduced two kinds of important information: the original attribute information of node itself (e.g., sequences of proteins, molecular fingerprints of drugs) and behavior information of the biomolecules. Then, a comparative experiment was carried out with a random forest (RF) classifier. The experiment results show that the behavior of the node contains more useful information than the attribute of the node in the DTIs prediction, and better results can be obtained.

## Results and Discussion

In order to illustrate that the behavior features of nodes contain more useful information than the traditional attribute features of biomolecules, we compared the performances of various well-known classifiers based on these two different types of features under five-fold cross-validation in various evaluation criteria. Cross-validation is mainly used to prevent over-fitting caused by over-complicated models. It is a statistical method used to evaluate the generalization ability of training data. For the five-fold cross-validation, the original data is randomly divided into five parts, and four parts are selected as the training set each time, and the remaining one part is used as the test set. The cross-validation was repeated five times, and the average value for the accuracy of the five runs was taken as the evaluation index of the final model. In this work, the number of the five training sets is 17,770, 17,770, 17,770, 17,770, 17,776, respectively; the number of five test sets is 4444, 4444, 4444, 4444, 4448, respectively.

### Performance Evaluation With Support Vector Machine on Two Different Features

In the experiment, we employed the state-of-the-art method Support Vector Machine (SVM) to assess the performance between the two different features on the integrated dataset. The two features include attribute features and behavior features. The attribute features are obtained from the molecular sequence information. The behavior features are derived from the MAN. We hypothesized that the MAN may assist in improving prediction performance. In order to ensure reasonable fairness, we set the same parameters to compare the performances of the two different features on the model. The results are shown in Tables 1, 2.

**TABLE 1:** Performance evaluation with SVM on attribute features.

5-folds	Acc (%)	TPR (%)	TNR (%)	PPV (%)	MCC (%)
1	66.16	66.79	65.53	65.96	32.32
2	66.22	66.16	66.29	66.25	32.45
3	66.49	67.64	65.35	66.12	33.00
4	67.06	67.69	66.43	66.84	34.12
5	66.74	67.37	66.11	66.53	33.49
Average	66.53 ± 0.37	67.13 ± 0.65	65.94 ± 0.48	66.34 ± 0.35	33.08 ± 0.75

**TABLE 2:** Performance evaluation with SVM on behavior features.

5-folds	Acc (%)	TPR (%)	TNR (%)	PPV (%)	MCC (%)
1	74.71	71.56	77.86	76.37	49.51
2	77.12	72.73	81.50	79.72	54.44
3	75.83	75.07	76.60	76.23	51.67
4	75.99	75.83	76.15	76.07	51.98
5	75.51	73.41	77.60	76.62	51.06
Average	75.83 ± 0.87	73.72 ± 1.73	77.94 ± 2.11	77.00 ± 1.53	51.73 ± 1.79

Meanwhile, receiver operating characteristic (ROC) curves are widely applied in many fields, such as machine learning, data mining, and so on. We also used ROC curves to measure the comprehensive index between the False Positive Rate and the True Positive Rate continuous variable. The area under curves (AUC) could be shown as the prediction accuracy of the classifier. The larger the AUC, the higher the accuracy.

**FIGURE 1:**
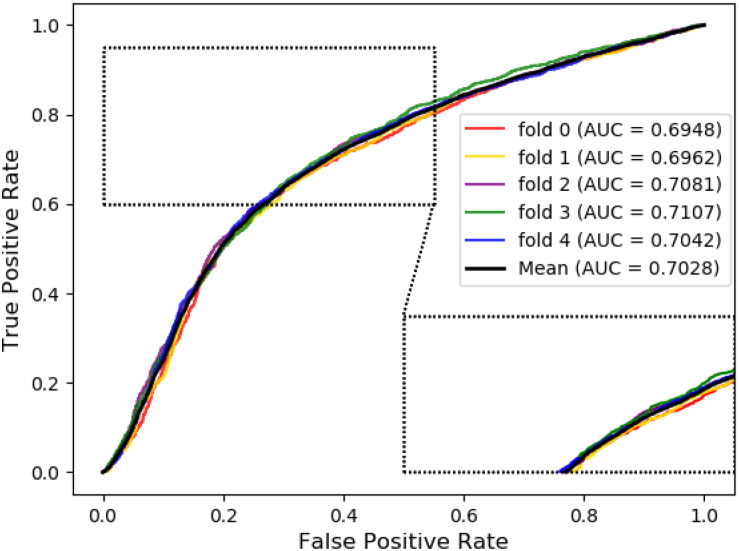
The ROC curve of SVM on attribute feature.

**FIGURE 2:**
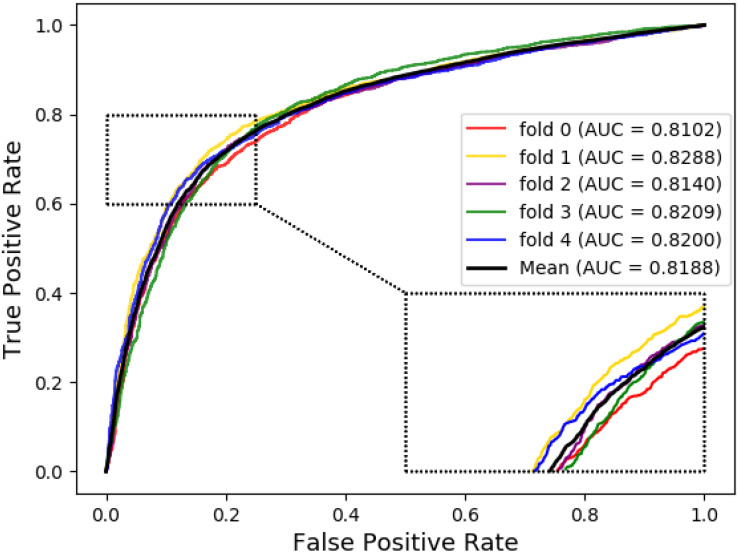
The ROC curve of SVM on behavior feature.

The ROC curve of the SVM classifier based on attribute feature and behavior feature with 5-fold cross-validation is shown in Figures 1, 2, respectively. It is clear that the average of AUC is 0.7028 by using attribute information, the average of AUC is 0.8188 by using behavior information based on MAN network. Hence, the behavior information of nodes play an important role in the DTIs predictions.

### Performance Evaluation With Random Forest on Two Different Features

In order to illustrate that the behavior features are indeed better than the attribute features, either on a single liner classifier or on an ensemble classifier, we also implemented the RF model on our experiment. In this experiment, we set the same parameters to compare the performances of the two different features on the model, the results are shown in Tables 3, 4.

**TABLE 3:** Performance evaluation with RF on attribute features.

5-folds	Acc (%)	TPR (%)	TNR (%)	PPV (%)	MCC (%)
1	81.37	77.59	85.15	83.93	62.92
2	81.98	78.62	85.33	84.27	64.10
3	81.80	79.16	84.43	83.56	63.68
4	80.49	76.78	84.20	82.94	61.15
5	80.71	76.30	85.13	83.69	61.67
Average	81.27 ± 0.66	77.69 ± 1.20	84.85 ± 0.50	83.68 ± 0.49	62.70 ± 1.27

**TABLE 4:** Performance evaluation with RF on behavior features.

5-folds	Acc (%)	TPR (%)	TNR (%)	PPV (%)	MCC (%)
1	85.58	79.93	91.22	90.11	71.61
2	86.16	80.38	91.94	90.89	72.81
3	85.76	80.56	90.95	89.9	71.9
4	84.18	77.63	90.73	89.33	68.96
5	85.56	79.86	91.26	90.13	71.58
Average	85.45 ± 0.75	79.67 ± 1.18	91.22 ± 0.46	90.07 ± 0.56	71.37 ± 1.44

**FIGURE 3:**
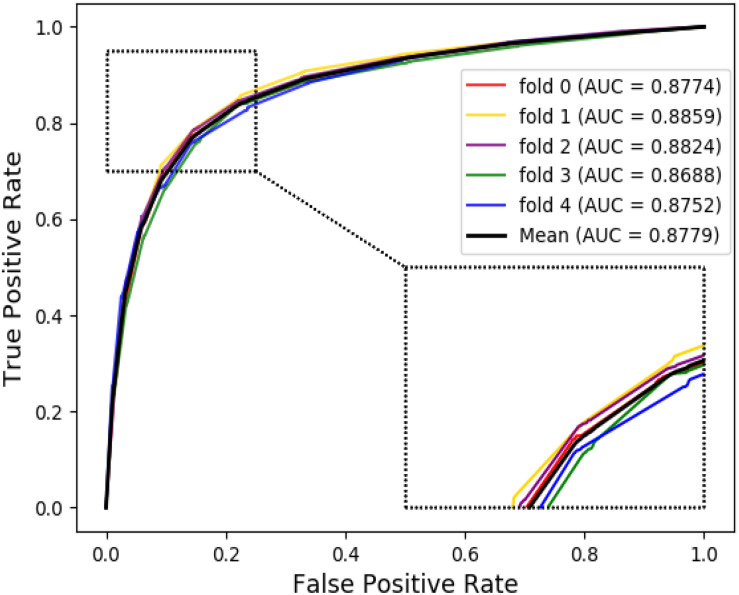
The ROC curve of random forest on attribute feature.

**FIGURE 4:**
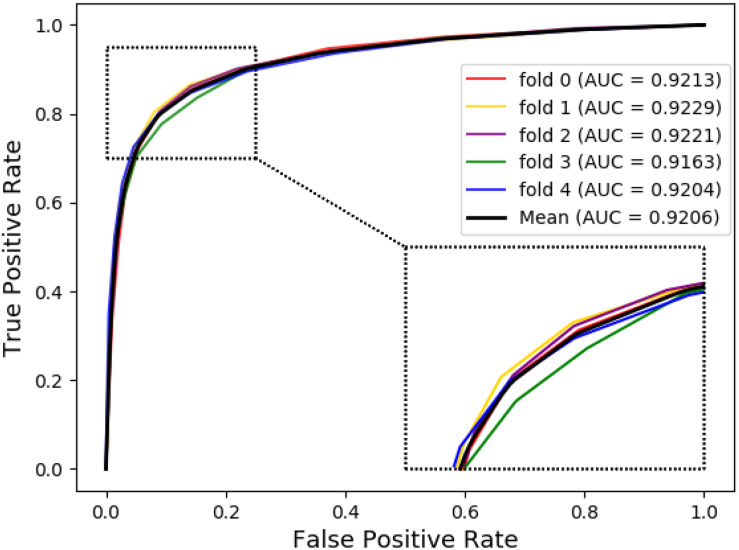
The ROC curve of random forest on behavior feature.

The ROC curves of the RF classifier based on attribute feature and behavior feature with five-fold cross-validation are shown in Figures 3, 4, respectively. It is obvious that the average of AUC is 0.8779 by using attribute information, the average of AUC is 0.9206 by using behavior information based on the MAN. So, the behavior information of nodes play an important role in the DTI predictions.

As mentioned above, it is apparent that the constructed MAN network can receive accurate DTI detection because more behavior information can be obtained from the complex biomolecular associations network. The presented complex network has made an indelible contribution to the prediction of DTIs. The main innovations can be summed up in the following two aspects: (1) Construction of the MAN network, which integrates five types of biomolecules and nine known relationships between them. It can provide a novel potential helpful tool for predicting new DTIs across the whole field of bioinformatics; (2) Behavior features were obtained by deep walk network embedding method, which can further optimize the performance of classifiers. This method can achieve more helpful information in the data than traditional attribute features. In a few words, experimental results revealed that our presented network is not only extremely suitable for DTI prediction, but also fit for other biomolecule associations prediction.

## Materials and Methods

### Datasets Construction

In this article, the heterogeneous data input to the MAN is collected from nine known relationships: DTIs, drug–disease associations (DDAs), protein–protein interactions (PPIs), protein–disease associations (PDAs), lncRNA–target interactions, protein–miRNA interactions, lncRNA–disease interactions, lncRNA–miRNA association, miRNA–disease association; which were shown in Table 5. These known relationships were also based on five types of biomolecules: drug, protein, disease, lncRNA, miRNA; which were listed in Table 6. The MAN contained topological relationships and distributions among all the molecules in the heterogeneous network. Considering the local and global connection modes, this work describes the basic context and intrinsic connection profiles for the whole nodes. Therefore, the prediction of DTIs can be determined by the connection relationships of the other nodes in the network.

**TABLE 5:** Nine known relationships in the molecular associations network.

Relationship	Database	Number
Drug–target	DrugBank (Wishart et al., 2017)	11107
Drug–disease	CTD (Davis et al., 2018)	18416
Protein–disease	DisGeNET (Piñero et al., 2016)	25087
lncRNA–target	LncRNA2Target (Cheng et al., 2018)	690
lncRNA–disease	LncRNADisease (Chen et al., 2012)	1264
	lncRNASNP2 (Miao et al., 2017)	
miRNA–target	miRTarBase (Chou et al., 2017)	4944
miRNA–disease	HMDD (Huang et al., 2018)	16427
miRNA–lncRNA	lncRNASNP2 (Miao et al., 2017)	8374
Protein–protein	STRING (Szklarczyk et al., 2016)	19237
Total	N/A	105546

**TABLE 6:** The number of 5 types of biomolecules from the nine known relationships.

Biomolecule	Number
Drug	1025
Target/Protein	1649
miRNA	1023
lncRNA	769
Disease	2062
Total	6528

### Multi-Molecular Network

**FIGURE 5:**
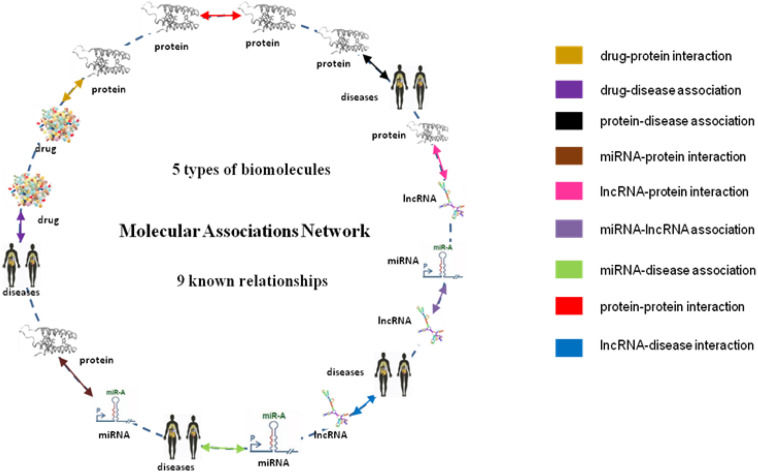
Construction of Multi-molecular Network.

From the collection of nine known relationships between five types of biomolecules annotated in many well-known databases which are mentioned above, we constructed a multi-molecular network, also called MAN by linking two arbitrary association nodes. The complex MAN is shown in Figure 5. Based on the known associations, some biomolecules are suggested to interact with each other. In the network graph, the heterogeneous nodes correspond to five types of biomolecules (drug, protein, disease, miRNA, and lncRNA), and edges correspond to associations among them. The construction of the systematic MAN network provides a new perspective for predicting interactions between drug and target.

### Traditional Attribute Representation

#### Drug Molecular Fingerprint

**FIGURE 6:**
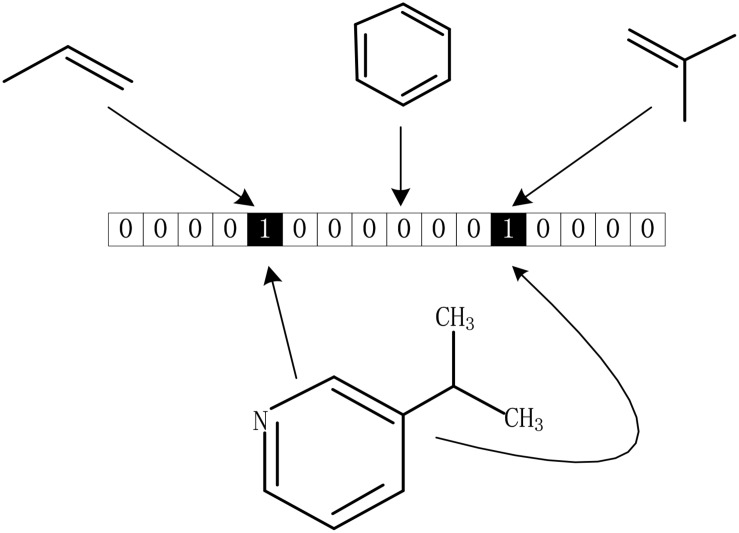
Representation of drug molecular fingerprint.

The drug molecular data was extracted from DrugBank database. To further process these data better, we calculated the Morgan fingerprints of drug molecules with the RDKit (Landrum, 2013) tool in python. The main idea of the molecular fingerprint method is that molecular structure is encoded as many substructure fingerprints in a series of binary bits, and a kernel is then applied to a molecule to generate a bit vector or count vector. Substructure pattern matching can be done using query molecules built from SMARTS which is first determined as a predefined dictionary (Guba et al., 2015). As we all know, there is a SMARTS-based implementation of the 166 public MACCS keys (Cereto-Massagué et al., 2015). As shown in Figure 6, each fingerprint bit corresponds to a fragment of the molecule, if its corresponding known fragment appears in the given molecule, the corresponding bit in the fingerprint is set to 1; otherwise, it is set to 0. Thus, each molecule can be represented as a Boolean array. In this method, although the whole molecule was divided into a great many of fragments, it still retains all the complexity of drug molecules.

#### Protein Sequence

The total protein sequence information was collected from the STRING database. For protein sequences, 20 types of amino acids were classified into four categories by the polarity of the side chain information, which contained (Ala, Val, Leu, Ile, Met, Phe, Trp, Pro), (Gly, Ser, Thr, Cys, Asn, Gln, Tyr), (Arg, Lys, His), and (Asp, Glu). Similarly, each protein sequence was transformed into a 64-dimensional (4 × 4 × 4) feature vector by counting the frequency of every subsequence appearing in the whole protein sequence, and each dimension of the vector is the normalized frequency of the corresponding 3-mer in the sequence (Rizk et al., 2013).

### Network Embedding—DeepWalk

In 2014, Perozzi et al. (2014) proposed DeepWalk, which can learn latent representation of vertices in a network. Analogous to word2vec, it uses the co-occurrence relationship among the whole nodes in the graph to learn the vector representation of nodes. There are two stages in the process of the deepwalk method: (1) A sequence of nodes is constructed. The locally associated training data is obtained by applying a random walk generator for sampling from each node in the homogeneous network. Then, to obtain a sequence for each node by imitating the process of text generation; (2) The Skip-Gram is used to train the sampling data, and the discrete nodes are represented as vectors in the network, and the Hierarchical Softmax is used to classify the ultra-large-scale classification.

#### Generation of Sequence of Nodes

**FIGURE 7:**
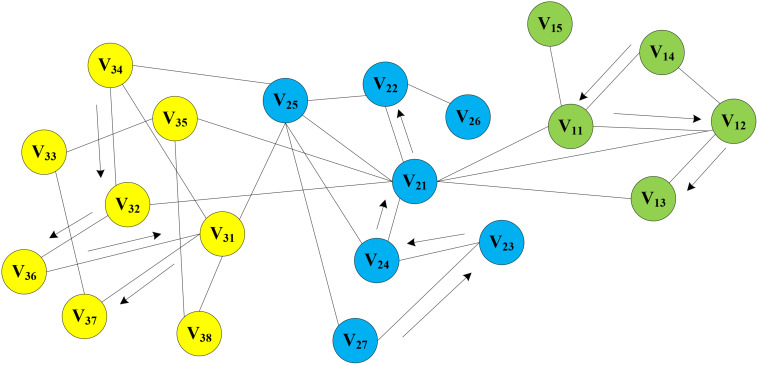
Random Walks on Molecular Associations Network.

In the MAN, a homogeneous network was constructed by five research objects (miRNA, lncRNA, drug, protein, and disease) at the cellular level. On the assumption that there is a network graph *G* a random vertex *v*_*i*_ is uniformly sampled as the root of the random walk. Then, a walk samples uniformly from each vertex to the adjacent nodes until it reaches the maximum length. In this way, the process of text generation is simulated to find sequence information for each node in the network, e.g., *V_14_->V_11_->V_12_->V_13_, V_27_->V_23_->V_24_->V_21_->V_22_, V_34_->V_32_->V_36_->V_31_->V_37_*, and so on. Random walks on MAN is shown in Figure 7. Afterward, the sequence of each node will be treated as a sentence in NLP as input of word2vec, and the vector representation of nodes is obtained.

#### Skip-Gram Model

Skip-Gram is one type of the word2vec model, which was proposed by McCormick (2016). It uses nodes to conjecture context, and learns vector representation by maximizing the co-occurrence probability of words within a window, and ignores the order in which nodes appear in sentences. The representation of nodes with the same context is similar. The higher the frequency of two nodes appearing in a sequence at the same time, the higher the similarity between the two nodes. The co-occurrence probability can be transformed into the product of conditional probability according to independence assumption, which can be summarized as follow:

(1) $$P\,\left(\frac{v_{i-c},\ldots ,v_{i+c}}{v_{i}|\Phi \,\left(v_{i}\right)}\right)=\prod_{\begin{array}{c} k=i-c\\ k\neq i \end{array}}^{i+c}P\,\left(v_{k}|\Phi \,\left(v_{i}\right)\right)$$

where, *v*_*i–c*_ and *v*_*i+c*_ are the left and right context of the word *v*_*i*_, c is the size of the window. In addition, we map each vertex *v*_*k*_ to its current representation vector *Φ*(*v*_*k*_)*∈ R^*d*^*.

The conditional probability of each vertex in the sequence is calculated, that is, the *log* value of the probability of other nodes in the sequence when the node appears, and the vector representation of the node is updated with the help of the stochastic gradient descent algorithm.

(2) $$J\,\left(\Phi \right)=-l\,o\,g\,P\,\left(u_{j}|\Phi \,\left(v_{k}\right)\right)$$

### Classification Models

Classification is one of the important tasks in data mining. The so-called classification is to classify the unknown data into existing categories according to its characteristics or attributes. That is to say, using given categories and known training data to learn classification rules and classifiers, and then predicting the unknown data.

#### Support Vector Machines

Support Vector Machine (SVM) is a supervised machine learning algorithm, which is mainly used for binary classification problems (Suykens and Vandewalle, 1999). In this algorithm, each data was considered as one point in *n*-dimensional space (*n* is the number of features), and each eigenvalue is a value of a specific coordinate. Then, classification is carried out by finding the hyper-planes that distinguish the two classes. In the sample space, the partition of hyper-planes can be described by the following linear equations:

(3) $$f\,\left(x\right)=w^{T}\,x+a=0$$

Assuming that it has completed the separation of samples and the labels of the two samples are {*+*1, −1}, for a classifier, *f*(*x*) > 0 represents the class that label is *+*1, otherwise, it is −1. In order to maximize the distance between the nearest two classes of samples on both sides of the plane, we need to find two hyper-planes parallel to and equal to the hyper-plane.

(4) $$f\,\left(x\right)=w^{T}\,x+a=+1$$

(5) $$f\,\left(x\right)=w^{T}\,x+a=-1$$

Then, to maximize the interval between these two hyper-planes *max*(*1/||w||*). Thus, SVM can provide a good generalization ability for classification problems.

#### Random Forest

Random forest is a relatively novel machine learning model. In the 1980s, Breiman (2017) developed the classification tree, which achieved classification and regression by repeating binary data, and the amount of calculation was greatly reduced. In 2001, Breiman combined classification trees into RFs, which randomized the use of variables (columns) and data (rows) to generate many classification trees, and then summarized the results of all the classification trees (Breiman, 2001). Random forest contains many decision trees in the forest, but there is no correlation between these trees. When a new sample is input to the forest, each decision tree will judge which category the sample should belong to. And then, the sample was predicted to be of the most selected category.

In the process of feature importance assessment using RF, it depends on the contribution of each feature to each tree in the RF. The contribution is usually measured by Gini index or error rate of out-of-bag (OOB) data. Assuming that there is *n* features *f_1_, f_2_, f_3_, …, f_*n*_*, the Gini variable importance measures (VIM) of each feature *f*_*i*_ can be described as follows:

(6) $$G\,i\,n\,i_{n}=\sum_{m=1}^{\left|M\right|}\sum_{m^{\prime }\neq m}p_{n\,m}\,p_{n\,m^{\prime }}=1-\sum_{m=1}^{\left|M\right|}p_{n\,m}^{2}$$

Where, *m* represents *m* classes. *p*_*nm*_ is the proportion of class *k* in node *n*.

### Performance Measurement Tools

In our study, in order to size up the effectiveness and steadiness of our constructed model, we counted the results of five parameters: Accuracy (Acc), recall (sensitivity, hit rate, or true positive rate (TPR), specificity (selectivity, or true negative rate (TNR), precision (positive predictive value (PPV) and Matthews’s Correlation Coefficient (MCC), respectively. These parameters can be represented as follows:

(7) $$A\,c\,c=\frac{T\,P+T\,N}{T\,P+F\,P+T\,N+F\,N}$$

(8) $$T\,P\,R=\frac{T\,P}{T\,P+F\,N}$$

(9) $$T\,N\,R=\frac{T\,N}{F\,P+T\,N}$$

(10) $$P\,P\,V=\frac{T\,P}{F\,P+T\,P}$$

(11) $$M\,C\,C=\frac{\left(T\,P\times T\,N\right)-\left(F\,P\times F\,N\right)}{\sqrt{\left(T\,P+F\,N\right)\times \left(T\,N+F\,P\right)\times \left(T\,P+F\,P\right)\times \left(T\,N+F\,N\right)}}$$

where *TP* is the count of true interacting pairs correctly predicted, i.e., the number of true positives. *FP* refers to the quantity of false positives, which is described as the number of true non-interacting pairs falsely predicted. *TN* means the quantity of true negatives, in other words, it represents the number of true non-interacting pairs predicted correctly. *FN* represents the quantity of false negatives, i.e., the true interacting pairs falsely predicted to be non-interacting pairs. According to these parameters, a Receiver Operating Characteristic (ROC) was plotted to evaluate the performance of the random projection method. Then we can calculate the AUC to assess the performance of the model.

## Conclusion

In this study, we investigated the relationship among drug, protein, miRNA, lncRNA and disease. Then, we developed a novel method to discover the potential interaction between drug and target on a large scale. We constructed a novel scheme based on the above five molecules and nine relationships arbitrarily between two molecules, which is called the MAN network. By focusing on this network, each node can obtain a feature vector by using node behavior information (the relationship of each node with others could be described by the deepwalk network embedding method). To our knowledge, this is the first report to predict DTIs from a complex heterogeneous network in an overall view at the cellular level. Experimental results demonstrated that our model has achieved good prediction results, which is a new attempt to predict DTIs. This work would have potential applications for drug discovery and repositioning.

## Data Availability Statement

The raw data required to reproduce these findings cannot be shared at this time as the data also forms part of an ongoing study. Requests to access the datasets should be directed to the corresponding author.

## Author Contributions

Z-HC and Z-HY conceived the algorithm, carried out analyses, prepared the data sets, carried out experiments, and wrote the manuscript. Z-HG and H-CY designed and performed the experiments. G-XL and Y-BW analyzed the experiments and checked the manuscript. All authors read and approved the final manuscript.

## Conflict of Interest

The authors declare that the research was conducted in the absence of any commercial or financial relationships that could be construed as a potential conflict of interest.

## References

[B1] Barabasi A.-L. Oltvai Z. N. (2004). Network biology: understanding the cell’s functional organization. *Nat. Rev. Geneti.* 5 101–113. 10.1038/nrg1272 14735121

[B2] Breiman L. (2001). Random forests. *Mach. Learn.* 45 5–32.

[B3] Breiman L. (2017). *Classification and Regression Trees*. Abingdon: Routledge.

[B4] Cahan P. Li H. Morris S. A. Lummertz da Rocha E. Daley G. Q. Collins J. J. (2014). CellNet: network biology applied to stem cell engineering. *Cell* 158 903–915. 10.1016/j.cell.2014.07.020 25126793 PMC4233680

[B5] Cereto-Massagué A. Ojeda M. J. Valls C. Mulero M. Garcia-Vallvé S. Pujadas G. (2015). Molecular fingerprint similarity search in virtual screening. *Methods* 71 58–63. 10.1016/j.ymeth.2014.08.005 25132639

[B6] Chan K. C. You Z.-H. (2016). “Large-scale prediction of drug-target interactions from deep representations,” in *Proceedings of the Neural Networks (IJCNN), 2016 International Joint Conference on*, Vancouver, BC: IEEE.

[B7] Chen G. Wang Z. Wang D. Qiu C. Liu M. Chen X. (2012). LncRNADisease: a database for long-non-coding RNA-associated diseases. *Nucleic Acids Res.* 41 D983–D986. 10.1093/nar/gks1099 23175614 PMC3531173

[B8] Chen X. Yan C. C. Zhang X. Zhang X. Dai F. Yin J. (2015). Drug–target interaction prediction: databases, web servers and computational models. *Brief. Bioinform.* 17 696–712. 10.1093/bib/bbv066 26283676

[B9] Cheng L. Wang P. Tian R. Wang S. Guo Q. Luo M. (2018). LncRNA2Target v2. 0: a comprehensive database for target genes of lncRNAs in human and mouse. *Nucleic Acids Res.* 47 D140–D144. 10.1093/nar/gky1051 30380072 PMC6323902

[B10] Chou C.-H. Shrestha S. Yang C. D. Chang N. W. Lin Y. L. Liao K. W. (2017). miRTarBase update 2018: a resource for experimentally validated microRNA-target interactions. *Nucleic Acids Res.* 46 D296–D302. 10.1093/nar/gkx1067 29126174 PMC5753222

[B11] Davis A. P. Grondin C. J. Johnson R. J. Sciaky D. McMorran R. Wiegers J. (2018). The comparative toxicogenomics database: update 2019. *Nucleic Acids Res.* 47 D948–D954. 10.1093/nar/gky868 30247620 PMC6323936

[B12] Devlin J. Chang W.-M. Lee K. Toutanova K. (2018). Bert: pre-training of deep bidirectional transformers for language understanding. *arXiv* [Preprint]. Available online at: https://arxiv.org/abs/1810.04805 (accessed May 24, 2019).

[B13] Emmert-Streib F. Glazko G. V. (2011). Network biology: a direct approach to study biological function. *Wiley Interdiscip. Rev. Syst. Biol. Med.* 3 379–391. 10.1002/wsbm.134 21197659

[B14] Goldberg Y. Levy O. (2014). word2vec explained: deriving Mikolov et al.’s negative-sampling word-embedding method. *arXiv* [Preprint]. Available online at: https://arxiv.org/abs/1402.3722 (accessed February 15, 2014).

[B15] Guba W. Meyder A. Rarey M. Hert J. (2015). *Torsion Library Reloaded: A New Version of Expert-Derived SMARTS Rules for Assessing Conformations of Small Molecules.* Washington, DC: ACS Publications. 10.1021/acs.jcim.5b00522 26679290

[B16] Guo Z.-H. Yi H.-C. You Z.-H. (2019). Construction and comprehensive analysis of a molecular association network via lncRNA–miRNA–Disease–Drug–Protein graph. *Cells* 8:866. 10.3390/cells8080866 31405040 PMC6721720

[B17] Heinemann F. Huber T. Meisel C. Bundschus M. Leser U. (2016). Reflection of successful anticancer drug development processes in the literature. *Drug Discov. Today* 21 1740–1744. 10.1016/j.drudis.2016.07.008 27443674

[B18] Huang Z. Shi J. Gao Y. Cui C. Zhang S. Li J. (2018). HMDD v3. 0: a database for experimentally supported human microRNA–disease associations. *Nucleic Acids Res.* 47 D1013–D1017. 10.1093/nar/gky1010 30364956 PMC6323994

[B19] Kitano H. (2002). Computational systems biology. *Nature* 420 206–210. 12432404 10.1038/nature01254

[B20] Knowles J. Gromo G. (2003). A guide to drug discovery: target selection in drug discovery. *Nat. Rev. Drug Discov.* 2 63–69. 10.1038/nrd986 12509760

[B21] Landrum G. (2013). Rdkit documentation. *Release* 1 1–79.

[B22] Lu Z.-L. Wang Y. Zeng M. Li M. (2019). “HNEDTI: prediction of drug-target interaction based on heterogeneous network embedding,” in *Proceedings of the 2019 IEEE International Conference on Bioinformatics and Biomedicine (BIBM)*, San Diego, CA: IEEE, 211–214.

[B23] Luo Y. Zhao X. Zhou J. Yang J. Zhang Y. Kuang W. (2017). A network integration approach for drug-target interaction prediction and computational drug repositioning from heterogeneous information. *Nat. Commun.* 8:573. 10.1038/s41467-017-00680-8 28924171 PMC5603535

[B24] Materi W. Wishart D. S. (2007). Computational systems biology in drug discovery and development: methods and applications. *Drug Discov. Today* 12 295–303. 10.1016/j.drudis.2007.02.013 17395089

[B25] Mayr A. Klambauer G. Unterthiner T. Steijaert M. Wegner J. K. Ceulemans H. (2018). Large-scale comparison of machine learning methods for drug target prediction on ChEMBL. *Chem. Sci.* 9 5441–5451. 10.1039/c8sc00148k 30155234 PMC6011237

[B26] McCormick C. (2016). *Word2vec Tutorial-the Skip-Gram Model.* Available online at: http://www.mccormickml.com.

[B27] Miao Y. R. Liu W. Zhang Q. Guo A. Y. Miao Y.-R. Liu W. (2017). lncRNASNP2: an updated database of functional SNPs and mutations in human and mouse lncRNAs. *Nucleic Acids Res.* 46 D276–D280. 10.1093/nar/gkx1004 29077939 PMC5753387

[B28] Pennington J. Socher R. Manning C. (2014). “Glove: global vectors for word representation,” in *Proceedings of the 2014 Conference on Empirical Methods in Natural Language Processing (EMNLP)*, Stroudsburg, PA: Association for Computational Linguistics.

[B29] Perozzi B. Al-Rfou R. Skiena S. (2014). “Deepwalk: online learning of social representations,” in *Proceedings of the 20th ACM SIGKDD International Conference on Knowledge Discovery and Data Mining*, New York, NY: ACM.

[B30] Peters M. E. Neumann M. Iyyer M. Gardner M. Clark C. Lee J. (2018). Deep contextualized word representations. *arXiv* [Preprint]. Available online at: https://arxiv.org/abs/1802.05365 (accessed March 22, 2018).

[B31] Piñero J. Bravo À Queralt-Rosinach N. Gutiérrez-Sacristán A. Deu-Pons J. Centeno E. (2016). DisGeNET: a comprehensive platform integrating information on human disease-associated genes and variants. *Nucleic Acids Res.* 45 D833–D883. 10.1093/nar/gkw943 27924018 PMC5210640

[B32] Rizk G. Lavenier D. Chikhi R. (2013). DSK: k-mer counting with very low memory usage. *Bioinformatics* 29 652–653. 10.1093/bioinformatics/btt020 23325618

[B33] Suykens J. A. Vandewalle J. (1999). Least squares support vector machine classifiers. *Neural Process. Lett.* 9 293–300. 10.1162/089976602753633411 11972910

[B34] Szklarczyk D. Morris J. H. Cook H. Kuhn M. Wyder S. Simonovic M. (2016). The STRING database in 2017: quality-controlled protein–protein association networks, made broadly accessible. *Nucleic Acids Res.* 45 D362–D368. 10.1093/nar/gkw937 27924014 PMC5210637

[B35] Wishart D. S. Feunang Y. D. Guo A. C. Lo E. J. Marcu A. Grant J. R. (2017). DrugBank 5.0: a major update to the DrugBank database for 2018. *Nucleic Acids Res.* 46 D1074–D1082. 10.1093/nar/gkx1037 29126136 PMC5753335

[B36] Wong Y. H. Lin C. L. Chen T. S. Chen C. A. Jiang P. S. Lai Y. H. (2015). Multiple target drug cocktail design for attacking the core network markers of four cancers using ligand-based and structure-based virtual screening methods. *BMC Med. Genomics* 8:S4. 10.1186/1755-8794-8-S4-S4 26680552 PMC4682379

[B37] Yamanishi Y. Kotera M. Kanehisa M. Goto S. (2010). Drug-target interaction prediction from chemical, genomic and pharmacological data in an integrated framework. *Bioinformatics* 26 i246–i254. 10.1093/bioinformatics/btq176 20529913 PMC2881361

[B38] Yang C. Liu Z. Zhao D. Sun M. Chang E. (2015). “Network representation learning with rich text information,” in *Proceedings of the Twenty-Fourth International Joint Conference on Artificial Intelligence*, Palo Alto, CA: AAAI Press.

[B39] Yang Z. Dai Z. Yang Y. Carbonell J. Salakhutdinov R. Le Q. V. (2019). XLNet: generalized autoregressive pretraining for language understanding. *arXiv* [Preprint]. Available online at: https://arxiv.org/abs/1906.08237.

[B40] Yildirim M. A. Goh K. I. Cusick M. E. Barabási A. L. Vidal M. (2007). Drug–target network. *Nat. Biotechnol.* 25 1119–1127. 17921997 10.1038/nbt1338

[B41] Zhang D. Yin J. Zhu X. Zhang C. (2018). Network representation learning: a survey. *IEEE Trans. Big Data* 6 3–28. 10.1109/TBDATA.2018.2850013

[B42] Zhang W. Lin W. Zhang D. Wang S. Shi J. Niu Y. (2019). Recent advances in the machine learning-based drug-target interaction prediction. *Curr. Drug Metab.* 20 194–202. 10.2174/1389200219666180821094047 30129407

